# Phase 3 Clinical Trial: Perioperative Use of Nonacog Gamma, a Recombinant Factor IX, in Previously Treated Patients With Moderate/Severe Hemophilia B

**DOI:** 10.1177/1076029620946839

**Published:** 2020-08-20

**Authors:** Jerzy Windyga, Margarita Timofeeva, Oleksandra Stasyshyn, Vasily Mamonov, José Luis Lamas Castellanos, Toshko Lissitchkov, Krzysztof Chojnowski, Miranda Chapman, Borislava G. Pavlova, Srilatha Tangada

**Affiliations:** 1 Department of Hemostatic Disorders and Internal Medicine, Laboratory of Hemostasis and Metabolic Diseases, 49564 Institute of Hematology and Transfusion Medicine, Warsaw, Poland; 2 Federal State Budgetary Institution of Science “Kirov Scientific and Research Institute of Hematology and Blood Transfusion of Federal Medico-Biological Agency,” Kirov, Russia; 3 Institute of Blood Pathology and Transfusion Medicine, Lviv, Ukraine; 4 National Research Center for Hematology, Moscow, Russia; 5 Hospital Dr. Sotero del Rio, Santiago, Chile; 6 National Center of Hematology, Sofia, Bulgaria; 7 Department of Hemostasis Disorders, Medical University of Lodz, Lodz, Poland; 8 Baxalta US Inc, a Takeda company, Cambridge, MA, USA; 9 Baxalta Innovations GmbH, a Takeda company, Vienna, Austria

**Keywords:** hemophilia B, previously treated patients, rFIX, BAX 326, nonacog gamma

## Abstract

Hemostatic management is essential for ensuring the safety of patients with hemophilia during surgery. This phase 3, prospective, uncontrolled trial, evaluated hemostatic efficacy, consumption, and safety of a recombinant factor IX concentrate, nonacog gamma (BAX 326, Rixubis^®^ [Baxalta US Inc., a Takeda company, Lexington, MA, USA]), in intraoperative and postoperative settings in previously treated patients (PTPs) with severe or moderately severe hemophilia B undergoing elective surgery (N = 38 surgeries; 21 major, 17 minor). Predefined preoperative hemostatic factor IX levels (80-100% of normal for major and 30-60% for minor surgeries) were maintained for each patient. Intraoperative efficacy was rated as “excellent” or “good” for all surgeries. Postoperative hemostatic efficacy on day of discharge was rated as “excellent,” “good,” and “fair,” respectively, for 29 (76.3%), 7 (18.4%), and 2 (5.3%) surgical procedures. All adverse events were considered unrelated to study drug; most frequently reported was mild procedural pain (9 patients). No thrombotic events, severe allergic reactions, or inhibitor formation were observed. Nonacog gamma was well tolerated and effective for intraoperative and postoperative hemostatic management of PTPs with hemophilia B.

NCT01507896, EudraCT: 2011-000413-39

## Introduction

Hemophilia B is characterized by coagulation system malfunction of the factor IX (FIX) gene, resulting in increased uncontrolled hemorrhages as well as severe hemarthroses and ensuing arthropathies.^1,2^ Increased life expectancy due to an expanding array of treatment options has placed patients with hemophilia at greater risk for developing hemophilic arthropathy as well as age-related comorbidities, often requiring cardiac, abdominal, or orthopedic surgical intervention. During invasive procedures, patients with hemophilia B require effective management of hemostasis by elevating FIX plasma activity to hemostatically sufficient levels prior to, during, and after surgery.^1,2^ However, due to low numbers of patients with hemophilia B,^3^ limited clinical data are available on the efficacy of conventional plasma-derived and recombinant coagulation FIX products used to maintain hemostasis during surgery.

Although a remote risk of infection remains for treatments containing albumin, recombinant replacement factor products are inherently free of blood-borne pathogens and could reduce the risk of potential transmission of adventitious infectious agents.^4^ The benefits of achieving adequate plasma levels of FIX using recombinant FIX in patients with hemophilia B are well known.^5–7^ A recombinant FIX, nonacog gamma (BAX 326, Rixubis^®^ [Baxalta US Inc., a Takeda company, Lexington, MA, USA]), with structural and functional characteristics comparable to endogenous FIX, was developed for the prevention and control of bleeding in patients with hemophilia B.^8–10^ Nonacog gamma is formulated in the absence of any human- or animal-derived components and is free of albumin. We have previously reported results of an interim analysis of this study after the first 10 major surgeries were completed.^11^ Here we present the final evaluation of the hemostatic efficacy, consumption, and safety of nonacog gamma in previously treated patients (PTPs) with severe or moderately severe hemophilia B undergoing surgery.

## Methods

### Study Design

This prospective, phase 3, open-label, uncontrolled, multicenter clinical study (https://ClinicalTrials.gov: NCT01507896, 5 protocol amendments; EudraCT: 2011-000413-39) was conducted at 10 clinical sites in 8 countries (Bulgaria, Chile, Columbia, Czech Republic, Poland, Romania, Russia, Ukraine) from December 19, 2011, to May 15, 2014.

### Participants

Eligible patients were aged 12 to 65 years, had severe (FIX level <1%) or moderately severe (FIX level 1-2%) hemophilia B, and were previously treated with plasma-derived and/or recombinant FIX concentrate(s) for a minimum of 150 exposure days, with no history of FIX inhibitors. Patients included in the study required surgical, dental, or other invasive procedures (emergency or elective, major or minor) and had participated in the pivotal (NCT01174446),^8^ pediatric (NCT01488994),^9^ or continuation (NCT01286779) nonacog gamma study. Patients who had not participated in a prior study could enroll only if they were planning to undergo major elective surgery. All patients provided written informed consent prior to enrollment.

### Study Objectives

The primary objective was to evaluate the hemostatic efficacy and safety of nonacog gamma in the intraoperative and postoperative setting in PTPs with severe (FIX level <1%) or moderately severe (FIX level 1-2%) hemophilia B undergoing major or minor elective or emergency surgical, dental, or other invasive procedures.

Intraoperative and postoperative hemostatic efficacy of nonacog gamma was rated by the operating surgeon and blood loss was determined, with consumption of nonacog gamma measured daily and as the total weight-adjusted dose. Safety was evaluated by monitoring for development of inhibitory and total binding antibodies to FIX, adverse events (AEs) related to nonacog gamma, and occurrence of thrombotic events.

### Surgical or Invasive Procedures

Surgeries were classified as “major” or “minor” according to the expected blood loss and potential requirement for additional therapy. Major surgeries^12,13^ were defined as those that involved moderate or deep sedation, general anesthesia, or major conduction blockade for patient comfort. These included major orthopedic (eg, joint replacement), major abdominal, intracranial, cardiovascular, spinal, and any other surgery that has a significant risk of large volume blood loss or blood loss into a confined anatomical space. Extraction of ≥2 teeth at the same surgery or extraction of the third molar were considered as major surgeries. Adeno-tonsillectomy was also considered as major surgery in children. Minor surgeries^12,13^ comprised surgeries that could be safely and comfortably performed on a patient who had received local or topical anesthesia, without more than minimal preoperative medication or minimal intraoperative sedation, and in which the likelihood of complications requiring hospitalization or prolonged hospitalization was remote. Examples of minor surgeries included interventions such as removal of skin lesions, arthroscopy, minor dental procedures, or dental extractions.

### Treatment

On the day of surgery, prior to the procedure, a blood sample was collected for determination of FIX activity and standard hematology parameters. Within 60 minutes of blood collection, patients were administered a preoperative bolus intravenous loading dose of nonacog gamma in order to elevate plasma FIX levels to a target peak (80-100% for major surgeries and 30-60% for minor procedures).^14,15^ Each loading dose was individually calculated according to the following formula that used the patient’s most recent incremental recovery measurement:

Required units = body weight (kg) × desired factor IX rise (%) (IU/dl) × {reciprocal of observed recovery}

Another blood sample was collected approximately 15 min after the loading dose for determination of FIX activity and activated partial thromboplastin time, to ensure target levels of FIX activity had been achieved.

The subsequent treatment regimen was determined in line with the standard of care. Following the loading dose, patients received nonacog gamma as a bolus infusion; the regimen determined by the intensity and duration of the hemostatic challenge. Supplemental doses of nonacog gamma could be given at the discretion of the investigator. For major surgeries, FIX activity was targeted to remain between 80% to 100% until wound healing, with dosing repeated every 8 to 24 hours, most commonly every 12 hours, depending on the patient’s individual pharmacokinetics (PK; half-life) until adequate wound healing, then therapy for at least another 7 days to maintain a FIX activity of 30% to 60% for 7 to 14 days. Presurgical PK was only determined in patients (n = 12) undergoing major elective surgery who had not undergone a PK assessment in the pivotal, pediatric or continuation nonacog gamma studies. For minor surgeries (eg, single dental extraction), FIX activity was maintained at 30% to 60%, with a dose frequency of approximately every 24 hours for ≥1 day. The dose regimens were supported by the standard clinical dose for marketed plasma-derived FIX concentrates used in the peri- and postoperative setting described in the literature,^14,15^ as well as guidelines provided by the Core Summary of Product Characteristics for human plasma derived and recombinant coagulation FIX products,^16,17^ by the World Federation of Hemophilia^17^ and literature reviews.^18^

Plasma FIX coagulation activity (1-stage clotting assay) and activated partial thromboplastin time were monitored every 24 hours, before and after infusion of nonacog gamma, to determine whether dose modifications were required.

### Hemostatic Efficacy

The hemostatic efficacy of nonacog gamma was assessed by the operating surgeon intraoperatively at drain removal or postoperatively day 3, and on the day of discharge, using ratings of “excellent,” “good,” “fair,” and “none” (Table 1).

**Table 1.** Rating Scale for Intra- and Postoperative Efficacy Assessment Criteria.

Intraoperative^a^ and Postoperative^b^ Efficacy	
Rating	Criteria
Excellent	Blood loss was less than or equal to that expected for the type of procedure performed (≤100%)
Good	Blood loss was up to 50% more than expected for the type of procedure performed (101-150%)
Fair	Blood loss was more than 50% of that expected for the type of procedure performed (>150%)
None	Uncontrolled hemorrhage that was the result of inadequate therapeutic response despite proper dosing, necessitating a change of FIX concentrate
Postoperative Efficacy Assessment 72 Hours Postoperatively^c^	
Rating	Criteria
Excellent	Postoperative hemostasis achieved with nonacog gamma was as good or better than that expected for the type of surgical procedure performed in a hemostatically normal patient
Good	Postoperative hemostasis achieved with nonacog gamma was probably as good as that expected for the type of surgical procedure performed in a hemostatically normal patient
Fair	Postoperative hemostasis with nonacog gamma was clearly less than optimal for the type of procedure performed but was maintained without the need to change the FIX concentrate
None	Patient experienced uncontrolled bleeding that was the result of inadequate therapeutic response despite proper dosing, necessitating a change of FIX concentrate

Abbreviation: FIX, factor IX.

^a^ Reflects the blood loss as compared with the expected amount of blood loss estimated preoperatively (by the operating surgeon) for the type of procedure in a hemostatically normal individual.

^b^ Reflects the volume in drain as compared with the volume estimated preoperatively (by the operating surgeon) for the type of procedure performed in a hemostatically normal individual.

^c^ In the case of major surgery and where no drain was employed, the postoperative hemostatic efficacy was to be assessed by the operating surgeon on postoperative day 3 (approximately 72 hours postoperatively).

The predicted intraoperative and postoperative blood loss (average and maximum volume) as expected for a hemostatically normal individual of comparable demographics and baseline characteristics was estimated by the surgeon prior to each intervention.^5,6^ Blood loss was measured by the total drainage fluid volume (if applicable), and blood loss into swabs and towels. Any bleeding episodes, associated treatments and blood transfusions were recorded throughout the perioperative period.

### Consumption

The daily and total weight-adjusted dose of nonacog gamma per patient was recorded from the initiation of surgery, until discharge from hospital: 1 to 3 days postoperatively for minor surgery and approximately 2 weeks postoperatively for major surgery.

### Safety

Safety was assessed by the incidence of AEs, development of inhibitory and total binding antibodies to FIX, and the occurrence of thrombotic events and severe allergic reactions, such as hives, pruritus, urticaria, erythema, angioedema, hypotension, pain or tightness in the chest, dizziness and/or dyspnea. Samples for inhibitory and total binding antibodies to FIX were taken at screening; on the day of discharge; and if the patient had excessive, unexplained bleeding at any time intraoperatively or postoperatively. The presence of FIX inhibitors was measured using the Nijmegen modification of the Bethesda assay, and total binding antibodies to FIX were determined using an enzyme-linked immunosorbent assay (ELISA).^19^

Safety assessments during the postoperative period and at study termination also included a daily evaluation for clinical signs of thrombosis. In case of clinical signs of thrombosis, additional diagnostic procedures were required according to each institution’s standard of care.

### Pharmacokinetics

Presurgical PK were assessed in patients aged ≥12 years and not previously enrolled in a prior trial or who did not have a PK assessment in the preceding trial. The samples for PK analysis were taken within 30 minutes before the start of the infusion and at 30 minutes, 6 hours, 24 hours, 48 hours, and 72 hours following the infusion. The PK parameters included area under the plasma concentration versus time curve from 0 to 72 hours postinfusion (AUC_0-72 h_/dose), area under the plasma concentration versus time curve from time 0 to infinity (AUC_0-inf_/dose), elimination phase half-life (t_1/2_), incremental recovery at 30 minutes postinfusion; mean residence time (MRT); clearance (CL); and volume of distribution at steady state (V_ss_).

### Statistical Analysis

Sample size was guided by the availability of patients participating in prior studies who required surgical procedures.^8,9^ No statistical considerations were applied to determine the sample size. The safety analysis set (SAS) comprised all patients exposed to nonacog gamma during the study. The full analysis set (FAS) comprised all patients exposed to nonacog gamma who provided data suitable for the hemostatic efficacy analysis. The per-protocol analysis set (PPAS) comprised all patients in the FAS who did not have protocol deviations that were associated with efficacy end points or serious breaches of protocol.

Descriptive statistics were used to evaluate the occurrence of product-related AEs and thrombotic events. Hemostatic efficacy was analyzed by the percentage of patients with an efficacy rating (“excellent,” “good,” “fair,” and “none”) at each time point. The differences between the expected and actual average and maximum blood loss were summarized using descriptive statistics including median and range. Noncompartmental methods were used to calculate PK parameters, which were summarized with descriptive statistics.

Data handling, including data quality assurance, complied with regulatory guidelines (eg, International Conference on Harmonized Good Clinical Practice) and standard operating procedures.

## Results

### Patients

**Figure 1.**
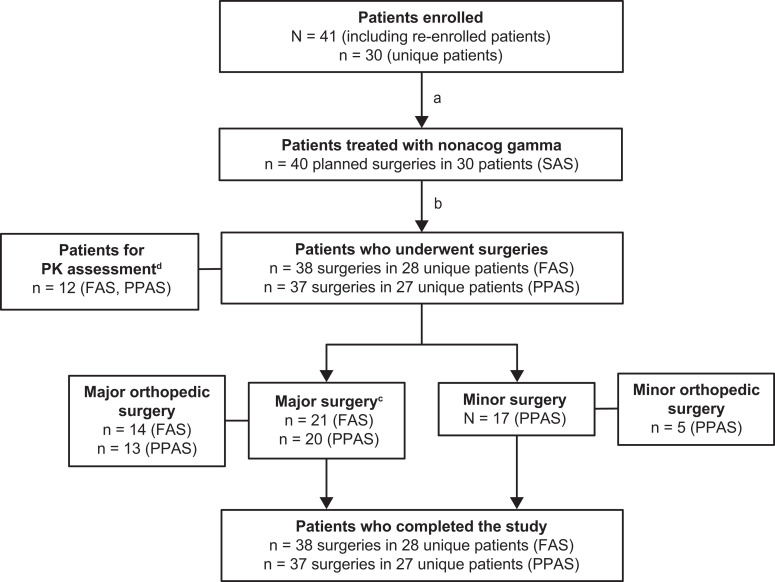
Patient flow and enrollments for surgery. Of 38 surgeries in 28 unique patients (FAS), 21 patients underwent a single surgery, 5 patients underwent 2 surgeries, 1 patient underwent 3 surgeries, and 1 patient underwent 4 surgeries. ^a^Consent was withdrawn by 1 patient. ^b^Consent was withdrawn by 1 patient; surgery denied for 1 patient. ^c^One major surgery excluded from the efficacy analysis, as FIX activity was not used to guide nonacog gamma dosing. ^d^Presurgical PK parameters were only assessed in patients who underwent major elective surgery, who had not undergone a PK assessment in a previous nonacog gamma study. Abbreviations: FAS, full analysis set; PPAS, per-protocol analysis set; SAS, safety analysis set.

There were 41 surgical enrollments in a study population of 30 unique patients, 8 of whom were enrolled for multiple surgeries (Figure 1). One patient discontinued the study before any treatment. The presurgical loading dose was administered for 40 surgeries. Safety analyses were performed for all surgeries that involved ≥1 dose of nonacog gamma (n = 40). Of the 40 planned surgeries, 21 were major and 17 were minor (FAS), and 2 were not performed. One major surgery was excluded from the PPAS, as FIX activity was not used to guide nonacog gamma dosing. All but 3 patients had participated in the pivotal or continuation nonacog gamma study.

Baseline characteristics of the study population were recorded in terms of enrollments for surgery and not by unique patients (Table 2). As a result, the number of treated patients (surgery and/or PK assessment) was higher (n = 40 in FAS/SAS; n = 39 in PPAS) than the number of unique patients who were included in this study (n = 30). Major surgeries (n = 21) included 14 orthopedic surgeries (11 joint replacements, 1 hardware/nail removal, and 2 synovectomies) and 7 non-orthopedic surgeries (3 abdominal, 3 dental, and 1 surgical excision of tumor from soft tissue). Minor surgeries (n = 17) comprised 5 orthopedic (4 intra-articular infiltration and 1 synoviorthesis) and 12 non-orthopedic surgeries (11 dental and 1 intra-articular injection).

**Table 2.** Baseline Characteristics of the Study Population by Surgical Enrollments.

Parameter	Statistic	Surgical Enrollments
Age at consent (years)	N	40
	Mean (SD)	39.7 (11.2)
	Median	41.5
	Range	17-57
Weight (kg)	N	40
	Mean (SD)	70.4 (11.0)
	Median	70.0
	Range	52-100
Gender	Male	40 (100.0)
	Female	0 (0.0)
FIX activity level (%)	<1%	18 (45.0)
	1-2%	11 (27.5)
	Not reported	11 (27.5)
FIX antigen level (%)	<1%	9 (22.5)
	≥1%	20 (50.0)
	1-2%	3 (7.5)
	>2-5%	2 (5.0)
	>5-<40%	4 (10.0)
	≥40%	11 (27.5)
	Not reported	11 (27.5)
Arthropathy at screening	Yes	39 (97.5)
	No	1 (2.5)
Type of surgery	Major	21 (52.5)
	Minor	17 (42.5)
	No surgery performed	2 (5.0)

Abbreviations: FIX, factor IX; SD, standard deviation.

### FIX Activity

The median (range) exposure to nonacog gamma on the day of surgery was 188.1 (124-296) IU/kg for major surgeries, and 88.1 (38-203) IU/kg for minor surgeries. The mean (standard deviation [SD]) FIX activity level (Table 3) for major surgeries was raised from 5.29% (5.40) to 110.80% ( 21.30) after the initial loading dose of nonacog gamma. During the first 3 postoperative days for major surgeries, the mean FIX levels, which were monitored every 24 hours for up to 11 days, increased from 55.08/62.00% (below the lower limit of the target FIX activity of 80%) to 117.44/121.62% after infusion of nonacog gamma (measured 10-30 minutes postinfusion), maintaining hemostatic efficacy in the postoperative setting. For minor surgeries, the mean (SD) FIX activity level was raised from 3.86% (2.67) to 67.54% (17.79) after the initial loading dose; subsequent FIX levels followed a similar pattern to the major surgeries on the first 3 postoperative days, with FIX levels increasing after 24 hours to hemostatic levels 10 to 30 minutes postinfusion (range: 65.65-78.02%) with nonacog gamma.

**Table 3.** Nonacog Gamma Pre- and Postinfusion Factor IX Activity Levels (% Normal).

Period	Time Point	Statistic*	Major	Minor	Orthopedic	Non-orthopedic
Day of surgery	60 min preoperative	n	21	16	18	19
		Mean (SD)	5.29 (5.40)	3.86 (2.67)	4.74 (5.04)	4.61 (3.92)
		Median	3.40	4.10	3.15	3.70
		Range	0.5-16.9	1.2-11.3	0.5-16.9	0.5-16.2
	15 min post bolus infusion	n	21	15	17	19
		Mean (SD)	110.80 (21.30)	67.54 (17.79)	102.77 (27.37)	83.83 (28.55)
		Median	108.70	65.20	106.30	79.20
		Range	65.1-142.7	49.2-115.6	49.2-141.7	49.5-142.7
Postoperative day 1	30 min preinfusion	n	21	17	19	19
		Mean (SD)	55.08 (16.91)	20.39 (10.52)	44.98 (25.26)	34.14 (18.55)
		Median	55.10	16.40	46.10	32.70
		Range	24.8-89.5	8.1-44.5	8.6-89.5	8.1-71.2
	10-30 min postinfusion	n	20	17	19	18
		Mean (SD)	117.44 (34.32)	65.65 (20.64)	105.10 (40.94)	81.55 (33.02)
		Median	118.65	62.00	106.10	69.20
		Range	58.5-176.2	31.6-110.8	37.1-176.2	31.6-168.5
Postoperative day 2	30 min preinfusion	n	21	8	14	15
		Mean (SD)	58.00 (16.96)	25.79 (8.89)	58.98 (15.37)	39.91 (21.74)
		Median	55.80	24.45	57.85	32.40
		Range	27.1-91.3	17.0-40.5	34.3-91.3	17.0-86.6
	10-30 min postinfusion	n	20	8	14	14
		Mean (SD)	117.66 (33.82)	65.84 (15.32)	123.78 (29.54)	81.93 (34.10)
		Median	120.05	62.25	124.65	67.80
		Range	65.5-172.0	50.6-98.4	70.1-172.0	50.6-158.1
Postoperative day 3	30 min preinfusion	n	21	5	14	12
		Mean (SD)	62.00 (18.26)	27.88 (12.18)	62.52 (16.84)	47.17 (24.78)
		Median	63.60	24.80	65.40	42.40
		Range	28.5-88.2	16.0-48.2	28.5-84.4	16.0-88.2
	10-30 min postinfusion	n	20	5	14	11
		Mean (SD)	121.62 (33.40)	78.02 (15.86)	130.88 (26.71)	90.01 (31.99)
		Median	130.00	84.90	133.30	84.90
		Range	57.5-164.3	57.6-95.1	69.9-164.3	57.5-154.2

Abbreviations: SD, standard deviation.

* Mean, median and range refer to factor IX activity levels (% normal).

### Hemostatic Efficacy

**Figure 2.**
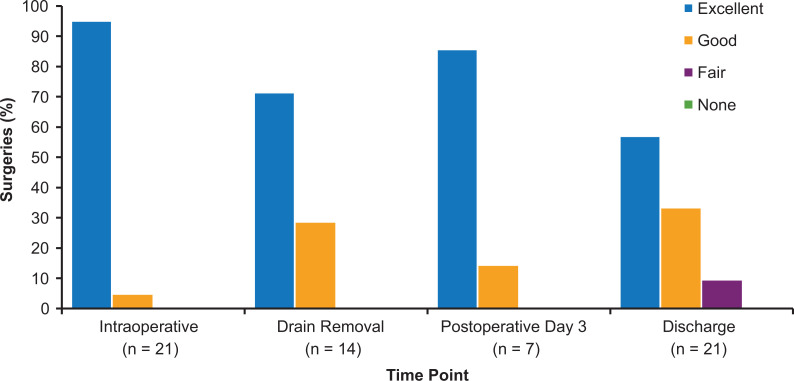
Perioperative hemostatic efficacy ratings in major surgeries.

For all surgeries (major and minor), the intraoperative hemostatic efficacy rating based on the 4-point scale (Table 1) was “excellent” (97.4%), except 1 knee joint replacement which had a rating of “good” (2.6%). At drain removal, the ratings for all surgeries with a drain placed (N = 14) was either “excellent” (n = 10; 71.4%) or “good” (n = 4; 28.6%). Two major surgeries (Figure 2) had an efficacy rating of “fair” at discharge (total left knee replacement and total right knee replacement); both had ratings of “excellent” intraoperatively and at drain removal. All minor surgeries received 100% “excellent” efficacy ratings, intraoperatively (n = 17), postoperatively at day 3 (n = 1), and at discharge (n = 17).

### Blood Loss

As expected, blood loss was higher in major than in minor surgeries (mean [SD]: 344.9 [SD = 420.1] mL vs 1.2 [SD = 1.1] mL). The mean difference from the predicted average blood loss (Table 4) was highest for major (n = 21; mean [SD]: −50.9 [SD = 213.0] mL, range: −600 to 200) and orthopedic surgeries (n = 19; mean [SD]: −58.3 [SD = 223.1] mL, range: −600 to 200).

**Table 4.** Actual and Difference From Predicted Average/Maximum Blood Loss.

Period	Parameter	Statistic	Major	Minor	Orthopedic	Non-orthopedic
Intraoperative	Actual blood loss (mL)^a^	n	21	17	19	19
		Mean (SD)	344.9 (420.1)	1.2 (1.1)	374.3 (432.1)	7.9 (12.7)
		Median	150.0	1.0	150.0	2.0
		Range	6-1400	0-3	0-1400	0-50
	Predicted average blood loss (mL)^b^	n	21	17	19	19
		Mean (SD)	294.0 (289.4)	3.6 (4.9)	316.1 (296.9)	12.2 (15.4)
		Median	150.0	2.0	300.0	5.0
		Range	7-800	1-20	1-800	1-50
	Difference from predicted average blood loss (mL)^c^	n	21	17	19	19
		Mean (SD)	-50.9 (213.0)	2.4 (4.9)	-58.3 (223.1)	4.2 (7.2)
		Median	0.0	1.0	0.0	1.0
		Range	-600 to 200	-1 to 20	-600 to 200	0 to 20
	Predicted maximum blood loss (mL)^b^	n	21	17	19	19
		Mean (SD)	566.9 (546.5)	13.8 (24.4)	605.9 (562.4)	32.9 (50.2)
		Median	300.0	4.0	600.0	10.0
		Range	10-1500	1-100	1-1500	2-200
	Difference from predicted maximum blood loss (mL)^c^	n	21	17	19	19
		Mean (SD)	222.0 (323.7)	12.5 (24.5)	231.6 (339.8)	25.0 (43.1)
		Median	100.0	3.0	100.0	5.0
		Range	-200 to 1000	1 to 100	-200 to 1000	0 to 170
Postoperative	Actual blood loss (mL)^a^	n	14	NA	12	2
		Mean (SD)	603.6 (388.7)	NA	695.0 (338.3)	55.5 (62.9)
		Median	545.0	NA	690.0	55.5
		Range	11-1270	NA	70-1270	11-100
	Predicted average blood loss (mL)^b^	n	14	NA	12	2
		Mean (SD)	382.2 (157.4)	NA	433.3 (91.3)	75.5 (105.4)
		Median	400.0	NA	425.0	75.5
		Range	1-600	NA	300-600	1-150
	Difference from predicted average blood loss (mL)^c^	n	14	NA	12	2
		Mean (SD)	-221.4 (331.7)	NA	-261.7 (342.8)	20.0 (42.4)
		Median	-180.0	NA	-245.0	20.0
		Range	-870 to 430	NA	-870 to 430	-10 to 50
	Predicted maximum blood loss (mL)^b^	n	14	NA	12	2
		Mean (SD)	750.8 (337.3)	NA	841.7 (253.9)	205.5 (275.1)
		Median	700.0	NA	900.0	205.5
		Range	11-1200	NA	500-1200	11-400
	Difference from predicted maximum blood loss (mL)^c^	n	14	NA	12	2
		Mean (SD)	147.1 (330.1)	NA	146.7 (353.1)	150.0 (212.1)
		Median	60.0	NA	60.0	150.0
		Range	-270 to 930	NA	-270 to 930	0 to 300

Abbreviation: NA, not applicable; SD, standard deviation.

^a^ Actual blood loss determined by drainage volume, if applicable, and the estimated blood loss into swabs and towels during the procedure.

^b^ Predicted average blood loss and predicted maximum blood loss as expected for a hemostatically normal individual was estimated by the surgeon prior to each intervention.

^c^ Predicted average/maximum blood loss minus actual blood loss.

The actual intraoperative blood loss in the majority of major surgeries was either below (n = 8) or equal to (n = 8) the predicted average blood loss. The blood loss volume for 5 major surgeries was higher than the predicted average. Of these, 2 were less than the predicted maximum blood loss, and 2 were equal to the predicted maximum blood loss. Only 1 surgery had an intraoperative blood loss that was higher than the predicted maximum blood loss (1400 mL vs 1200 mL). In minor surgeries, intraoperative blood loss was below the predicted average for 12/17 minor surgeries; there were no cases of blood loss higher than the maximum predicted for a hemostatically normal individual.

For the 4/14 major surgeries (all orthopedic: joint replacement, removal of residual nail from femur fracture, knee replacement, hip replacement) with an actual postoperative blood loss exceeding the predicted maximum blood loss, FIX levels on postoperative days 1 to 3 were in the lower range of target FIX levels for 2 patients (34.3-40.2% and 40.2-56.5%). One patient, whose postoperative blood loss exceeded the maximum predicted by 10 mL, had FIX levels within the recommended range on postoperative days 1 to 3 (84.4-91.3%); this patient had a drain placed, which was not planned at the time the blood loss was estimated. The fourth patient exceeded the maximum predicted postoperative blood loss by 100 mL; this patient had FIX levels ranging from 55.6 to 81.1% on postoperative days 1 to 3.

Five patients, each of whom underwent major orthopedic surgery, received blood product transfusions in the form of packed red blood cells, fresh frozen plasma, or both (2-5 infusions; mean [SD]: 2.8 [1.3] units); 1 of these patients also received packed red blood cells during the postoperative period.

### Bleeding Episodes

Bleeding episodes were reported for 2 patients. Both patients (aged 45 and 43 years and both with severe chronic knee arthropathy) had undergone major orthopedic surgery (knee replacements) and had minor bleeding from the surgical incision at 7.9 and 9.9 days after surgery and at 2.6 and 3.2 hours after the last nonacog gamma infusion, respectively. Hospitalization was not required in either case.

### Consumption

All major surgeries (n = 21) utilized daily doses of nonacog gamma from the day of surgery until postoperative day 5. Fifteen surgeries continued to utilize daily doses of nonacog gamma until postoperative day 11 and beyond (up to postoperative day 28). Of the 19 orthopedic surgeries, 12 utilized daily doses of nonacog gamma from the day of surgery through postoperative day 11 and beyond. On the day of surgery, the mean (SD; range) weight-adjusted dose (IU/kg) for major surgery (n = 21) was 191.5 (50.6; 124-296), and 87.2 (42.9; 38-203) for minor surgery (n = 17).

During the postoperative period, the mean (SD; range) weight-adjusted dose (in IU/kg) for major surgery (n = 21) was 1350 (617; 415-2965), and 138 (136; 38-601) for minor surgery (n = 17).

### Safety

Nonacog gamma was well tolerated during perioperative management in PTPs with hemophilia B. None of the patients developed inhibitors or total binding antibodies to FIX. There were no severe allergic reactions and no thrombotic events. No deaths or treatment-related serious AEs occurred. Thirty-nine nonserious AEs were reported for 15/40 (37.5%) instances of nonacog gamma administration. One case of moderate hemorrhagic anemia was initially considered to be “possibly related” to treatment with nonacog gamma. The patient, for whom this AE was reported, went into surgery (a total left knee replacement) with a very high FIX activity level (105.8 IU/dL); FIX levels were kept constantly high throughout the entire postoperative period, which is a prerequisite for stable hemostasis. Therefore, this AE was subsequently assessed as unrelated to nonacog gamma, and related to the surgical procedure. The most frequently reported AE was procedural pain (15 reports for 9 patients, all considered “mild”). Thrombocytosis was reported 4 times (for 4 patients) and was considered “mild” in 3 patients and “moderate” in 1 patient. Postprocedural swelling was reported 4 times (for 4 patients) and was also considered “mild” in 3 patients and “moderate” in 1 patient. Abnormal hematology results such as thrombocytosis were either considered a consequence of the surgery or “not clinically significant” or due to a pre-existing disease.

### Pharmacokinetic Parameters

The presurgical PK parameters determined for 12 patients (Table 5) were similar to those obtained in the pivotal study (NCT01174446).^8^

**Table 5.** Presurgical Pharmacokinetic Parameters of Nonacog Gamma.

PK Parameter (N = 12)	Mean (SD)	Median (Range)
AUC_0-72 h_/dose (IU·hr/dL: IU/kg)	18.48 (6.43)	16.94 (11.64-37.26)
AUC_0-inf_/dose (IU·hr/dL: IU/kg)	20.60 (7.32)	19.24 (12.91-42.17)
MRT (hours)	27.17 (4.03)	27.33 (20.74-34.63)
CL (dL/[kg·hour])	0.0523 (0.0126)	0.0520 (0.0237-0.0775)
IR at 30 minutes postinfusion (IU/dL: IU/kg)	1.00 (0.29)	0.98 (0.64-1.73)
C_max_ (IU/dL)	77.43 (22.47)	75.70 (47.70-134.40)
Half-life (hours)	23.60 (3.60)	23.43 (17.76-29.60)
V_ss_ (dL/kg)	1.41 (0.38)	1.43 (0.70-2.06)

Abbreviations: AUC_0-72 h_/dose, area under the plasma concentration versus time curve from 0 to 72 hours postinfusion; AUC_0-inf_/dose, area under the plasma concentration versus time curve from time 0 to infinity; CL, clearance; C_max_, corresponds with the concentration of factor IX at ∼30 minutes; IR, incremental recovery; MRT; mean residence time; and V_ss_, volume of distribution at steady state.

## Discussion

A FIX concentrate therapy that provides the optimal dose and administration rate is essential for patients with hemophilia B in the management of bleeding during high-risk surgical settings and can lead to a positive postoperative clinical outcome.

Nonacog gamma was well tolerated and hemostatic efficacy was demonstrated for perioperative management in 38 surgeries during this first controlled, prospective, multicenter clinical study in a surgical setting of PTPs (aged 12-65 years) with severe and moderately severe hemophilia B undergoing major or minor elective or emergency surgical, dental, or other invasive procedures.

During the first 3 postoperative days, the mean preinfusion FIX levels increased from 55.08/62.00% to 117.44/121.62% for major surgeries, and from 20.39/27.88% to 65.65/78.02% for minor surgeries postinfusion with nonacog gamma, maintaining hemostatic efficacy in the postoperative setting. Thus, when used for perioperative management, nonacog gamma allows for achieving and maintaining recommended postoperative FIX levels (80-100% for major surgeries and 30-60% for minor surgeries), which is consistent with the European Agency for the Evaluation of Medicinal Products, Committee for Proprietary Medicinal Products (CPMP) guidelines.^17^ This differs slightly from guidance provided by the World Federation of Hemophilia,^20^ which recommends initial trough levels of 60% to 80% for major surgeries to be maintained for ≥3 days.

Overall, this study met its primary efficacy end point, and the final outcome is consistent with that reported for other recombinant coagulation factors used for intraoperative and postoperative management in individuals with hemophilia B.^5,6,15^ Intraoperatively, hemostasis was rated as “excellent” (97.4%) in all surgeries except 1 knee joint replacement, which had a rating of “good” (2.6%). At discharge from hospital, 76.3% of surgeries were rated “excellent” and 18.4% were “good.” Two surgeries, both major (total left knee replacement and total right knee replacement), had an efficacy rating of “fair” at discharge from hospital, following a rating of “excellent” intraoperatively and at drain removal.

In general, intraoperative and postoperative blood loss in the study was similar to that expected for the type of invasive procedures in patients without hemophilia undergoing surgery.^5,6^ Intraoperative actual blood loss was below or equal to the maximum predicted blood loss for all but 1 surgery. As reported previously, the mean postoperative blood loss was equal to or above the maximum predicted blood loss for 4 major surgeries, in which 3 patients had lower postoperative FIX levels than targeted and 1 patient received an unplanned drain, which was not considered at the time of blood loss estimation.^7,11^ The lower postoperative FIX levels seen in these 3 patients could be explained by increased clearance of FIX during/after surgery,^21^ as well as increased FIX consumption due to activation of hemostasis by tissue damage and blood loss.^22^

Limitations to this study include small sample size, and the heterogeneity of surgeries (extent of trauma, extent and duration of blood loss perioperatively). Direct comparisons (eg, blood loss, consumption) were not possible due to several factors, such as the difference in surgical settings and standards, preinfusion FIX levels, use of tourniquets and drains, and wide variability in blood loss levels per patient (Table 4). In this study, the intraoperative and postoperative blood loss for major surgeries following treatment with nonacog gamma ranged from 6 to 1400 mL and 11 to 1270 mL respectively, indicating the role of multifactorial conditions that influence postoperative blood loss.

There were no deaths or serious AEs reported for any of the patients who were treated with nonacog gamma for perioperative management. In addition, there were no severe allergic reactions, no bleeding complications, no thrombotic events, and no FIX inhibitor formation or binding antibody development observed during the study. Overall, nonacog gamma had a favorable safety profile when used in major or minor elective or emergency surgical, dental, or other invasive procedures for perioperative management in PTPs with severe to moderately severe hemophilia B.

When used for perioperative management, nonacog gamma allows for maintenance of postoperative FIX levels as suggested by CPMP^17^ for major and minor surgeries, and provides a reliable, convenient treatment option for patients with hemophilia B, allowing for appropriate intraoperative and postoperative hemostatic control.

## References

[1] Castaldo G Nardiello P Bellitti F, et al. Haemophilia B: from molecular diagnosis to gene therapy. Clin Chem Lab Med. 2003;41(4):445–451. 12747585 10.1515/CCLM.2003.067

[2] Mannucci PM Tuddenham EG The hemophilias—from royal genes to gene therapy. N Engl J Med. 2001;344(23):1773–1779. 11396445 10.1056/NEJM200106073442307

[3] Uprichard J Adamidou D Goddard NJ Mann HA Yee TT Factor IX replacement to cover total knee replacement surgery in haemophilia B: a single-centre experience, 2000-2010. Haemophilia. 2012;18(1):46–49. 21545378 10.1111/j.1365-2516.2011.02552.x

[4] Di Minno G Perno CF Tiede A, et al. Current concepts in the prevention of pathogen transmission via blood/plasma-derived products for bleeding disorders. Blood Rev. 2016;30(1):35–48. 26381318 10.1016/j.blre.2015.07.004 PMC7115716

[5] Roth DA Kessler CM Pasi KJ, et al. Human recombinant factor IX: safety and efficacy studies in hemophilia B patients previously treated with plasma-derived factor IX concentrates. Blood. 2001;98(13):3600–3606. 11739163 10.1182/blood.v98.13.3600

[6] White G Shapiro A Ragni M, et al. Clinical evaluation of recombinant factor IX. Semin Hematol. 1998;35(2 suppl 2):33–38. 9565165

[7] Windyga J Abbuehl BE Hafeman AE BAX326 (recombinant coagulation factor IX) for the treatment and prophylaxis of hemophilia B. Expert Rev Hematol. 2014;7(3):333–342. 24832133 10.1586/17474086.2014.903153

[8] Windyga J Lissitchkov T Stasyshyn O, et al. Pharmacokinetics, efficacy and safety of BAX326, a novel recombinant factor IX: a prospective, controlled, multicentre phase I/III study in previously treated patients with severe (FIX level <1%) or moderately severe (FIX level </=2%) haemophilia B. Haemophilia. 2014;20(1):15–24. 10.1111/hae.12228 23834666

[9] Urasinski T Stasyshyn O Andreeva T, et al. Recombinant factor IX (BAX326) in previously treated paediatric patients with haemophilia B: a prospective clinical trial. Haemophilia. 2015;21(2):196–203. 25495591 10.1111/hae.12548

[10] Windyga J Solano Trujillo MH Hafeman AE BAX326 (RIXUBIS): a novel recombinant factor IX for the control and prevention of bleeding episodes in adults and children with hemophilia B. Ther Adv Hematol. 2014;5(5):168–180. 25324957 10.1177/2040620714550573 PMC4199092

[11] Windyga J Lissitchkov T Stasyshyn O, et al. Efficacy and safety of a recombinant factor IX (BAX326) in previously treated patients with severe or moderately severe haemophilia B undergoing surgical or other invasive procedures: a prospective, open-label, uncontrolled, multicentre, phase III study. Haemophilia. 2014;20(5):651–658. 24697870 10.1111/hae.12419

[12] American College of Surgeons. Guidelines for office-based surgery. Published 2003 Accessed June 3, 2019 http://www.massmed.org/Physicians/Legal-and-Regulatory/Office-Based-Surgery-Guidelines/

[13] Australian Haemophilia Centre Directors’ Organisation. Guideline for the management of patients with haemophilia undergoing surgical procedures. Published 2010 Accessed June 3, 2019 http://www.ahcdo.org.au/documents/item/13

[14] Chowdary P Dasani H Jones JA, et al. Recombinant factor IX (BeneFix) by adjusted continuous infusion: a study of stability, sterility and clinical experience. Haemophilia. 2001;7(2):140–145. 11260272 10.1046/j.1365-2516.2001.00494.x

[15] Ragni MV Pasi KJ White GC, et al. Use of recombinant factor IX in subjects with haemophilia B undergoing surgery. Haemophilia. 2002;8(2):91–97. 11952843 10.1046/j.1365-2516.2002.00587.x

[16] Committee for Proprietary Medicinal Products. Core SPC for human plasma derived and recombinant coagulation factor IX products. Published 2000 Accessed June 3, 2019 https://www.ema.europa.eu/en/documents/scientific-guideline/guideline-core-spc-human-plasma-derived-recombinant-coagulation-factor-ix-products_en.pdf

[17] Committee for Medicinal Products for Human Use. Guideline on core SPC for human plasma derived and recombinant coagulation factor IX products—rev. 1. Published 2007 Accessed June 3, 2019 https://www.ema.europa.eu/en/documents/scientific-guideline/draft-guideline-core-spc-human-plasma-derived-recombinant-coagulation-factor-ix-products_en.pdf

[18] Hermans C Altisent C Batorova A, et al. Replacement therapy for invasive procedures in patients with haemophilia: literature review, European survey and recommendations. Haemophilia. 2009;15(3):639–658. 19444969 10.1111/j.1365-2516.2008.01950.x

[19] Whelan SF Hofbauer CJ Horling FM, et al. Distinct characteristics of antibody responses against factor VIII in healthy individuals and in different cohorts of hemophilia A patients. Blood. 2013;121(6):1039–1048. 23243272 10.1182/blood-2012-07-444877

[20] Wiedel J Stabler S Geraghty S Funk S Joint replacement surgery in hemophilia. Published 2010 Accessed June 3, 2019 http://www1.wfh.org/publication/files/pdf-1210.pdf

[21] Hoots WK Leissinger C Stabler S, et al. Continuous intravenous infusion of a plasma-derived factor IX concentrate (Mononine) in haemophilia B. Haemophilia. 2003;9(2):164–172. 12614367 10.1046/j.1365-2516.2003.00721.x

[22] Hazendonk H Preijers T Liesner R, et al. Perioperative replacement therapy in haemophilia B: an appeal to “B” more precise. Haemophilia. 2018;24(4):611–618. 29707861 10.1111/hae.13469

